# The value of DCE-MRI in assessing histopathological and molecular biological features in induced rat epithelial ovarian carcinomas

**DOI:** 10.1186/s13048-017-0362-z

**Published:** 2017-09-26

**Authors:** Su Juan Yuan, Tian Kui Qiao, Jin Wei Qiang, Song Qi Cai, Ruo Kun Li

**Affiliations:** 1 Department of Oncology, Jinshan Hospital, Shanghai Medical College, Fudan University, 1508 Longhang Road, Shanghai, 201508 People’s Republic of China; 2 Department of Radiology, Jinshan Hospital, Shanghai Medical College, Fudan University, 1508 Longhang Road, Shanghai, 201508 People’s Republic of China; 3 Department of Radiology, Zhongshan Hospital, Shanghai Medical College, Fudan University, 180 Fenglin Road, Shanghai, 200032 People’s Republic of China

**Keywords:** Epithelial ovarian carcinoma (EOC), Dynamic contrast-enhanced magnetic resonance imaging, Microvessel density, Vascular endothelial growth factor, Cancer antigen 125

## Abstract

**Background:**

To investigate dynamic contrast enhanced magnetic resonance imaging (DCE-MRI) for assessing histopathological and molecular biological features in induced rat epithelial ovarian carcinomas (EOCs).

**Methods:**

7,12-dimethylbenz[A]anthracene (DMBA) was applied to induce EOCs in situ in 46 SD rats. Conventional MRI and DCE-MRI were performed to evaluate the morphology and perfusion features of the tumors, including the time-signal intensity curve (TIC), volume transfer constant (K^trans^), rate constant (K_ep_), extravascular extracellular space volume ratio (V_e_) and initial area under the curve (IAUC). DCE-MRI parameters were correlated with histological grade, microvascular density (MVD), vascular endothelial growth factor (VEGF) and fraction of Ki67-positive cells and the serum level of cancer antigen 125 (CA125).

**Results:**

Thirty-five of the 46 rats developed EOCs. DCE-MRI showed type III TIC more frequently than type II (29/35 vs. 6/35, *p* < 0.001) in EOCs. The two types of TIC of tumors had significant differences in the histological grade, MVD, expression of VEGF and Ki67, and the serum level of CA125 (all *p* < 0.01). K^trans^, K_ep_ and IAUC values showed significant differences in different histological grades in overall and pairwise comparisons except for IAUC in grade 2 vs. grade 3 (all *p* < 0.01). There was no significant difference in V_e_ values among the three grade groups (*p* > 0.05). K^trans^, K_ep_ and IAUC values were positively correlated with MVD, VEGF and Ki67 expression (all *p* < 0.01). V_e_ was not significantly correlated with MVD, VEGF expression, Ki67 expression and the CA125 level (all *p* > 0.05).

**Conclusions:**

TIC types and perfusion parameters of DCE-MRI can reflect tumor grade, angiogenesis and cell proliferation to some extent, thereby helping treatment planning and predicting prognosis.

## Background

Imaging examination provides an important basis for diagnosing, staging malignant cancer and making rational treatment plans. In addition, it plays a vital role in predicting treatment response and monitoring recurrence. Magnetic resonance imaging (MRI) has been considered the preferred imaging modality for gynecologic disease due to its multidirectional and multi-parameter imaging characteristics and exquisite soft-tissue resolution [1]. Conventional MRI is only helpful in determining the location, size and nature of the ovarian lesion, and evaluating the response to treatment [2]. As a newly developed method, DCE-MRI quantitatively analyzes the tumor angiogenesis, vascular permeability and spatial distribution of the contrast agent, and has demonstrated the value in predicting disease free survival, prognostic factors, antitumor effects in breast, liver and prostate [3–7].

However, latest study showed that semi-quantitative DCE-MRI had performed poorly when distinguishing malignant from borderline ovarian tumors [8]. Up to date, the value of quantitative DCE-MRI for investigating histological grades of EOCs has not been performed. Therefore, the aim of our study was to investigate whether quantitative DCE-MRI is a useful tool for assessing histopathological and molecular biological features in induced rat epithelial ovarian carcinomas (EOCs) by correlating with histopathological grades, microvascular density (MVD), vascular endothelial growth factor (VEGF) and Ki67 expressions and the serum level of cancer antigen 125 (CA125).

## Methods

### Animals and the rat EOC model

This study was approved by Institutional Review Board of Jinshan Hospital of Fudan University and performed in strict accordance with the Guide for the Care and Use of Laboratory Animals of the National Science and Technology Committee of China. Fourty-six female SD rats (8 weeks old), Shanghai Laboratory Animal Research Center, Shanghai, China) were fed a standard diet and were housed in pathogen-free facilities at 25 ± 1 °C. The right ovary of each rat was surgically exposed, packed a cloth strip (5 mm × 5 mm) coated 2 mg 7,12-dimethylbenz[A] anthracene (DMBA, Sigma Chemical Company, St. Louis, MO, USA), and closed with the surround fatty substance. Benzylpenicillin injection of 10^5^ units was administered intraperitoneally to prevent infection before the abdominal wall was closed. The surgical procedures were referred to previous study [9]. Based on our preliminary experiment, rat EOCs in situ model were successfully established at 180 days after DMBA exposure.

### Imaging protocol

One hundred and eighty days after DMBA induction, MRI was carried out using a 3.0 T scanner (Verio, Siemens Healthcare, Erlangen, Germany) and a coil specially designed for rats. First, routine axial T1- and T2-weighted imagings with and without fat faturation were performed to detect the lesions. Second, DCE-MRI using fast low angle shot-2D (FLASH 2D) T1WI with fat saturation (TR/TE, 7.92 ms/2.28 ms) was performed in axial, sagittal and coronal planes after the caudal intravenous administration of 0.2 mmol/kg gadopentetate dimeglumine (Gd-DTPA, Magnevist; Bayer Schering, Berlin, Germany) and at a rate of 0.3 ml/s. A total of 40 phases of images were sequentially acquired with a temporal of 6 s. The scanning parameters were as follows: slice thickness = 1.0 mm, flip angle = 15°, gap = 1 mm, matrix size = 224 × 370, field of view = 80 mm × 62.5 mm and excitation = 4. The total scan duration was 4 min.

### Processing and analysis of the quantitative parameters of DCE-MRI

Using Tissue 4D software (Siemens, Erlangen, Germany) and two-compartment (Tofts) modeling, the DCE-MRI images were evaluated by two blinded radiologists each with 6 years of experience in pelvic MRI [10]. The values of the image signal intensity were measured in the slice with a maximum diameter of ovary lesions. By avoiding vessels and flow artifacts as much as possible, three circular regions of interest (ROI) (20–50 mm^2^) were selected in the solid areas of every lesion, achieved time-signal intensity curves (TICs) and generating the following quantitative parameters automatically: 1) volume transfer constant, K^trans^; 2) reverse volume transfer constant, K_ep_; and 3) extravascular extracellular space (EES) volume per unit volume of tissue, Ve (the relationship among these three parameters was as follows: K_ep_ = K^trans^/V_e_) [11]; and 4) initial areas under the time-intensity curve, IAUC. According to the study of Thomassin-Naggara [12], TIC was divided into three types:type I was defined as a gradual increase without a well-defined shoulder; type II was defined as a moderate initial increase relative to that of myometrium followed by a plateau; type III was defined as an initial increase steeper followed by a plateau.

### ELISA

At target time point, rats were immunized by intraperitoneal injection of 10% chloral hydrate, and blood was then taken from the heart. After centrifugation, the supernatants were collected and analyzed for the evaluation of cancer antigen 125 (CA125) in murine serum by ELISA (R&D Systems, Minneapolis, MN) according to the manufacturer’s instructions.

### Histopathological and immunohistochemical analysis

After MRI scanning, all rats were sacrificed, dissected and examined for gross abnormalities. Macroscopical specimen of entire reproductive tracts were harvested from the rats and fixed in 10% formalin. The specimen was dissected into sections at 1-3 mm interval by L.W., with 10 years of experience in human and murine gynecological pathology. The tumors were analyzed for morphology, and then the tissues were embedded in paraffin and cut into 3-μm-thick slices. Hematoxylin-eosin (HE) staining was performed to observe the histological type. Immunohistochemical (IHC) (Primary antibodies of CD31, VEGF and Ki67 purchased from Cell Signaling Technology, CA, USA) was applied to investigate the MVD, expression of VEGF and Ki67 in positive cells in EOCs. MVD was analyzed in CD31-stained vascular endothelial cells and determined as the mean microvessel at 3 microscopic fields of vision (HPF, × 200). VEGF expression displayed as brownish yellow granules in the cytoplasm and intercellular spaces, and determined as the mean integrated optical density (IOD) measured by Image-Pro Plus image analysis software at 3 HPFs (×200). Ki67-positive expression indicated the appearance of yellow or brown granules in the nucleus and determined as the percentage of positive tumor cells in 1000 tumor cells assessed at 10 HPFs (×200) with 1000 random cells.

### Statistical analysis

Data were analyzed using SPSS 22.0, and values were represented as the means ± standard deviation. Analysis of variance was used for data analysis between multiple groups, and the values between every two groups were analyzed by independent sample T test. The ordinal date was analyzed by rank sum test. Pearson’s correlation was used to analyze the relationship between the DCE-MRI parameters and VEGF, MVD, Ki67 and CA125 expression as follows: a correlation coefficient between 0.75 and 1.00 was considered highly relevant, that between 0.50 and 0.74 was considered moderately relevant, that between 0.25 and 0.49 was considered weakly relevant and that ≤0.249 was not considered relevant. [13]. For T test and rank sum test, *p* < 0.05 was considered significant; for correlation analysis, *p* < 0.01 was considered significant.

## Results

### Successful establishment of the rat ovarian cancer model

Based on previous study and our preliminary experiment, there was best result of induced EOCs and survival rates at 180 days after DMBA implantation. Fourty-four of 46 rats survived and 42 of 44 developed ovarian lesions. All cases were confirmed by postoperative pathology: 1 cyst, 1 cystadenoma, 35 EOCs (19 serous adenocarcinomas, 9 mucous, 4 mixed, 2 endometrioid, 1 clear cell carcinoma) and 5 other non-epitholial malignancies, Thirty-five EOCs accounted for 87.5% of all malignant ovarian tumors. Thirty-five EOCs accounted for 87.5% of all ovarian malignancies, The maximum diameter of tumors ranged from 15.2 ~ 31.0 mm with mean diameter of 25.5 ± 0.76 mm. Tumors appeared as cystic in 6 cases, solid-cystic in 25 cases and solid in 4 cases on conventional MRI and were proven by pathology.

### Histological grades of different TIC types in induced EOCs

DCE-MRI showed none of type I(0%) TIC, 6 type II (17.14%), and 29 type III (82.86%), with statistical significance (*p*<0.001). Compared with tumors with curve type II TIC, tumors with type III showed significantly higher histological grades (*p*<0.01, Table 1).

**Table 1:** Histological grades in 35 induced EOCs with different TIC types

Histological grade (WHO)	TIC II	TIC III
Grade 1	4 (40.0%)	6 (60.0%)
Grade 2	1 (11.1%)	8 (88.9%)
Grade 3	1 (6.2%)	15 (93.8%)

### DCE-MRI parameters in different histological grades of 35 induced EOCs

The mean K^trans^ value was 0.22 ± 0.06, mean K_ep_ value was 0.48 ± 0.13, mean V_e_ value was 0.51 ± 0.12 and mean IAUC value was 27.62 ± 8.15 in 35 rat EOCs. DCE-MRI parameters in different histological grades are summarized in Table 2. The mean K^trans^, K_ep_, and IAUC values significantly increased along with tumor grades increasing. There were significant differences in the overall comparison and in pairwise comparisons (all *p* < 0.05) except for IAUC in grade 2 vs. grade 3. There was no significant difference in the mean V_e_ values among the three grades (*p* > 0.05).

**Table 2:** Comparisons of DCE-MRI parameters in different histological grades in 35 induced EOCs

Histological grade	K^trans^	K_ep_	V_e_	IAUC
Grade 1	0.16 ± 0.01	0.36 ± 0.02	0.51 ± 0.02	20.08 ± 1.86
Grade 2	0.22 ± 0.01^c^	0.47 ± 0.03^c^	0.53 ± 0.02	28.07 ± 1.96^c^
Grade 3	0.26 ± 0.01^ab^	0.55 ± 0.02 ^ab^	0.53 ± 0.02	32.33 ± 1.91^b^

*p* < 0.05 ^a^: grade3 vs. grade 2; ^b^: grade 3 vs. grade 1; ^C^: grade 2 vs. grade 1

### MVD, VEGF and Ki67 expressions, and CA125 level EOCs with different TIC types

**Fig. 1:**
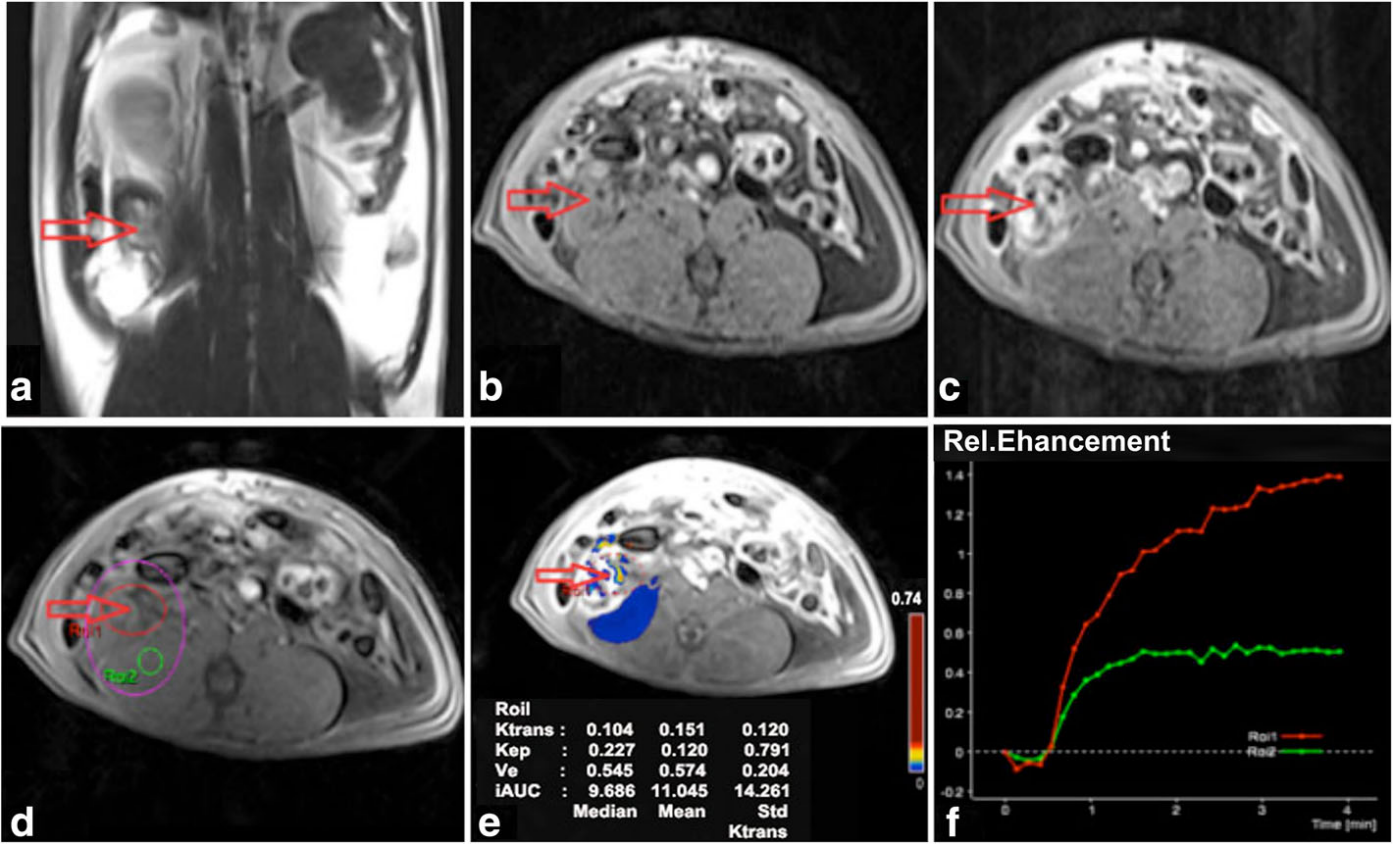
DCE-MRI imaging of EOCs with TIC II. In the right adnexal area, a multilocular cystic-solid was observed on T2WI with fat suppression (**a**) and T1WI (**b**) on the coronal position, showing obvious enhancement and solid portion on contrast-enhanced T1WI with fat suppression (**c**). In DCE-MRI images, ROI, including ROI1 (ovarian lesion) and ROI2 (muscle), were selected (**d**); pseudo-color images can be obtained by quantitatively analyzing DCE-MRI (**e**); the dynamic curve appeared as a moderate initial increase followed by a plateau (**f**). The red arrow points to the tumor focus

**Fig. 2:**
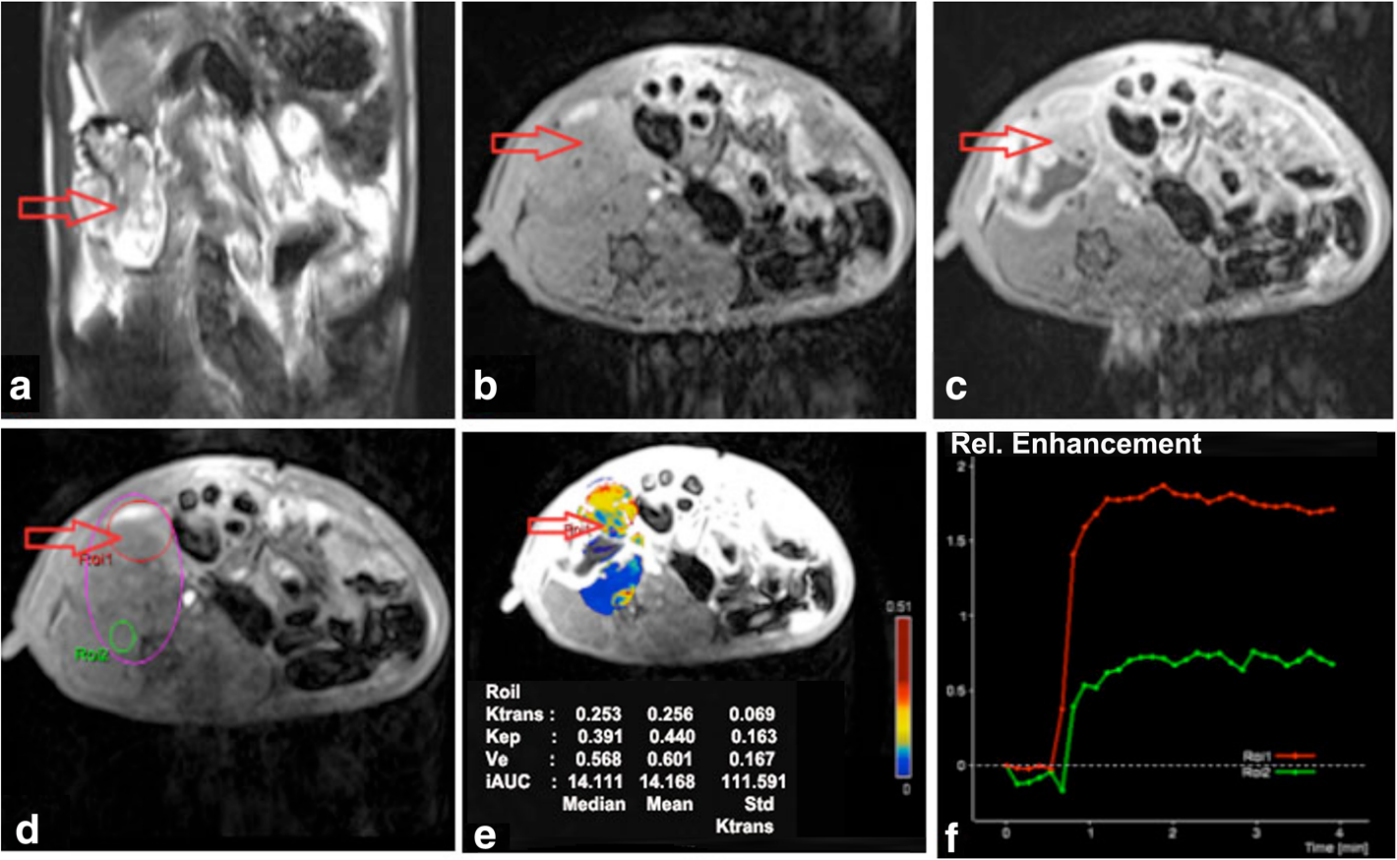
DCE-MRI imaging of EOCs with TIC III. In the right adnexal area, a multilocular cystic solid was observed on T2WI with fat suppression (**a**) and T1WI (**b**) on the coronal position, showing obvious enhancement and a solid portion on contrast-enhanced T1WI with fat suppression (**c**). In DCE-MRI images, ROI, including ROI1 (ovarian lesion) and ROI2 (muscle), were selected (**d**); pseudo-color images can be obtained by quantitatively analyzing DCE-MRI (**e**); the dynamic curve appeared as an initial steeper increase followed by a plateau (**f**). The red arrow points to the tumor focus

**Fig. 3:**
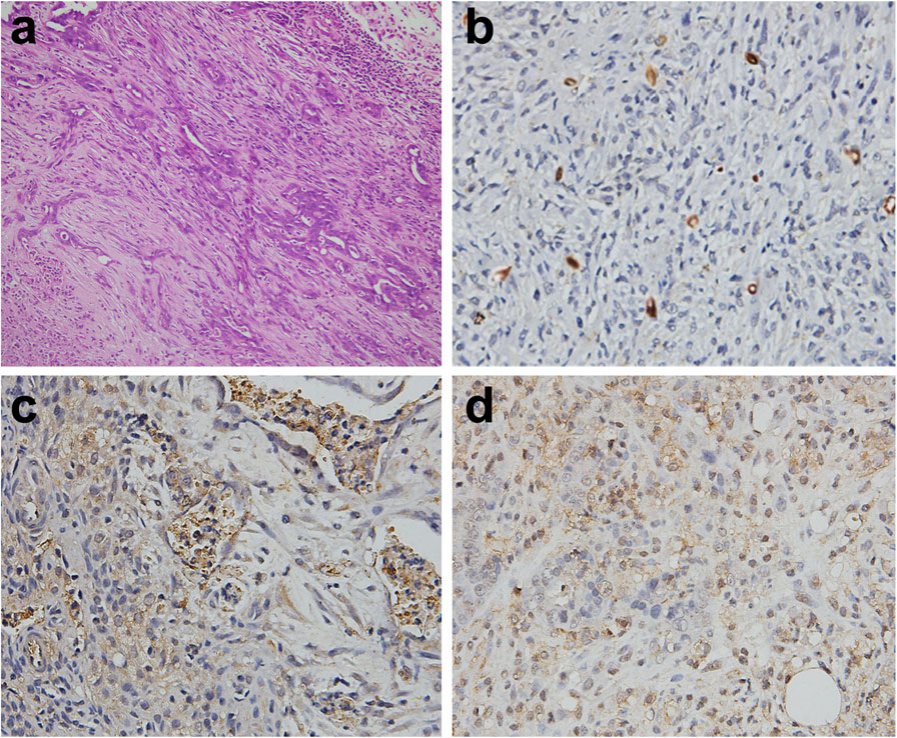
Pathological observation of EOCs with TIC II. H&E staining of (grade 2) EOCs demonstrated incomplete gland lumens, and tumor cells had presented a derangement distribution, and heterotypic cells with partially dark-stained nuclei have been observed (**a**). Brown microvessels with CD31 staining were observed with IHC method (**b**). IHC staining showed VEGF-positive expression displayed brownish yellow granules in the cytoplasm and intercellular spaces (**c**). IHC staining displayed yellow or brown granules appeared in Ki67-positive cell nuclei (**d**). (×200)

**Fig. 4:**
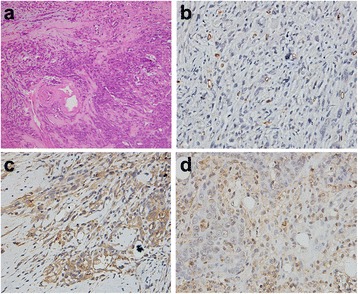
Pathological observation of EOCs with TIC III. H&E staining of (grade 3) EOCs demonstrated that gland lumens had disappeared, tumor cells had larger nucleus and more distinct nucleoli (**a**). Brown microvessels with CD31 staining were observed with IHC method (**b**). IHC staining showed VEGF-positive expression displayed brownish yellow granules in the cytoplasm and intercellular spaces (**c**). IHC staining displayed yellow or brown granules appeared in Ki67-positive cell nuclei (**d**). (×200)

As shown in Table 3, the MVD, expressions of VEGF and Ki67-positive tumor cells and CA 125 level between tumors with type II and type III TIC showed statistical significance (*p*<0.01) (Figs. 1, 2, 3 and 4).

**Table 3:** Comparisons of MVD, VEGF and Ki67 expression and CA125 level in different TIC types in induced EOCs

	TIC II	TIC III	t	*p*
MVD	20.17 ± 1.81	24.1 ± 3.4	2.672	0.012
VEGF (×10^3^)	(14.46 ± 7.40)	(35.79 ± 17.00)	2.987	0.005
Ki67 (%)	(22.2 ± 13.1)	(37.5 ± 11.1)	4.649	0.005
CA 125(U/mL)	(242.17 ± 104.29)	(353.44 ± 65.60)	3.848	0.009

### Correlations between DCE-MRI parameters and MVD, VEGF expression and Ki67-positive expression and CA125

As shown in Table 4, the mean K^trans^ was highly positively correlated with MVD (23.46 ± 3.602) and VEGF ((32.13 ± 17.68) × 10^3^) and was moderately positively correlated with the Ki67-positive cell proportion (34.91 ± 12.70) % and CA125 level (33.13 ± 82.17) U/mL (all *p*<0.01); K_ep_ was moderately positively correlated with MVD, VEGF, Ki67 and weakly positively correlated with CA125 (all *p*<0.01); no significant correlation was observed between V_e_ and MVD, VEGF, Ki67 or CA125 (all *p*>0.05); IAUC was highly positively correlated with VEGF, moderately positively correlated with MVD and Ki67, and weakly positively correlated with CA125 (all *p*<0.01).

**Table 4:** Correlation between the DCE-MRI parameters and MVD, VEGF and Ki67 expression and CA125 level

	K^trans^		K_ep_		V_e_		IAUC	
	r	*p*	r	*p*	r	*p*	r	*p*
MVD	0.755	<0.001	0.502	0.002	0.146	0.463	0.714	<0.001
VEGF	0.808	<0.001	0.526	0.001	0.181	0.287	0.752	<0.001
Ki67	0.689	<0.001	0.537	0.001	0.071	0.686	0.670	<0.001
CA125	0.541	0.001	0.448	0.007	0.042	0.809	0.486	0.003

## Discussion

EOCs makes up 85%–90% of ovarian maligancies, is one of the three major gynecological malignant tumors in the world and its mortality rate ranks first [14]. Poor prognosis may be associated with inaccurate staging, being diagnosed at late stages, easy recurrence and metastases after operation, drug-resistance and improper choice of antineoplastic drug. Therefore, accurate staging and evaluating histopathological and molecular biological characteristics of EOCs pre-treatment contributes to the rational choice of treatment strategy and prognosis prediction [15].

In this study, with 180 days DMBA-induction continuously, we successfully established rat EOCs in situ, of which EOCs accounted for 87.5% of all ovarian malignancies. These results demonstrated histological similarities to human ovarian malignancies, which would be suitable for DCE-MRI study. Based on the induced rat EOCs model, our study showed the value of DCE-MRI in assessing histopathological and molecular biological features of tumors by correlation between TIC, quantitative parameters (K^trans^, K_ep_ and IAUC) and histopathological grade, MVD, VEGF and Ki-67 expressions, and CA125 level.

Higher-grade ovarian cancer has a higher potency in terms of angiogenesis [16]. MVD is substitute biomarker of tumor angiogenesis [17]. VEGF is the most characteristic natural precursors of angiogenic factors [18]. Ki-67 is considered the most reliable marker for detecting the proliferative activity of tumor cells [19]. CA125 is an important indicator to diagnose ovarian cancer and is associated with differentiation and progression [20]. MVD, VEGF and its receptor, Ki67 and CA125 might affect the outcome of patients with malignant tumors, including EOCs [21–25].

TIC has been applied in the differential diagnosis of breast, prostate, ovary and other lesions, suggesting that all benign tumors had type I, the majority of malignant tumors had type III, only a small number of them had type II, and none had type I; type III TIC appeared to be specific for malignant tumors and was never observed in benign tumors [26, 27]. Our study showed that type III TIC outnumbered type II considerably, which was in accordance with the previous literatures [28, 29]. Moreover, compared with EOCs with curve type II TIC, EOCs with type III demonstrated significantly higher histological grade, MVD, VEGF expression, Ki67-positive cells and serum CA125 level, indicating a poor prognosis due to its high but immature neovascularization, low differentiation and active proliferative capacity.

K^trans^ and K_ep_ reflect vessel permeability and tissue perfusion, as well as tumor angiogenesis. Our results showed that K^trans^ and V_e_ increased significantly along with higher histological grade, and were positively correlated with MVD, VEGF expression, the fraction of Ki67-positive cells and the CA125 level. The higher the histological grade of, more active proliferation and more neovascularization in malignant tumors signify, the more incomplete basement membrane, leading to higher vascular permeability, exchanges of contrast agent consequently, and higher K^trans^ and K_ep_ values. Our findings were also in agreement with previous findings [30, 31].

IAUC is associated with blood flow in the tumor, volume and interstitial space, and is the comprehensive reflection of the changes in K^trans^, K_ep_ and V_e_ [32]. In our study, compared with that in grade 1, IAUC in grade 2 and 3 increased dramatically, which suggested that IAUC could reflect histological grade of EOCs to a certain extent. IAUC was positively correlated with MVD, VEGF expression, the fraction of Ki67-positive cells and CA125 level. We considered that the more neovascularization, higher permeability, together with, densely packed tumor cells and confined interstitial space in higher grades of EOCsresulted in increased IAUC value.

Ve represents the indirect appearance of tumor angiogenesis to some extent. In our findings, no significant relationships were observed between V_e_ and histological grade, MVD, VEGF expression, the fraction of Ki67-positive cells and the CA125 level. The reason might be that, on the one hand, more neovascularization and higher permeability increased blood perfusion in tumor tissue, resulting in a larger proportion of EES with contrast agent; on the other hand, an increasing vessels and tumor cells can result in a relatively decreased EES.

## Conclusions

In conclusion, our results revealed that the TIC types and parameters of DCE-MRI, K^trans^, K_ep_ and IAUC, could reflect the histological grade, angiogenesis, and cell proliferation to some degree, thereby helping treatment planning, predicting prognosis and recurrence, as well as guide targeted drug therapy in the clinical setting. However, the specific threshold values and how they are related to patient’s physical constitution and clinical staging need further investigation.

## Abbreviations

CA125: Cancer antigen 125
DCE-MRI: Dynamic contrast enhanced magnetic resonance imaging
DMBA: 7, 12-dimethylbenz[A]anthracene
EOCs: Epithelial ovarian carcinomas
H&E staining: Hematoxylin-eosin staining
IHC: Immunohistochemical
MVD: Microvascular density
TIC: Time-signal intensity curve
VEGF: Vascular endothelial growth factor
